# OpenHands: An Open-Source Statistical Shape Model of the Finger Bones

**DOI:** 10.1007/s10439-024-03560-7

**Published:** 2024-07-03

**Authors:** T. A. Munyebvu, C. D. Metcalf, C. B. Burson-Thomas, D. Warwick, C. Everitt, L. King, A. Darekar, M. Browne, M. O. W. Heller, A. S. Dickinson

**Affiliations:** 1 University of Southampton, Southampton, UK; 2 University Hospital Southampton NHS Foundation Trust, Southampton, UK

**Keywords:** Principal component analysis, Anatomy modelling, Machine Learning, Proximal interphalangeal joint, Distal interphalangeal joint, Anthropometrics, Ergonomics

## Abstract

This paper presents statistical shape models of the four fingers of the hand, with an emphasis on anatomic analysis of the proximal and distal interphalangeal joints. A multi-body statistical shape modelling pipeline was implemented on an exemplar training dataset of computed tomography (CT) scans of 10 right hands (5F:5M, 27–37 years, free from disease or injury) imaged at 0.3 mm resolution, segmented, meshed and aligned. Model generated included pose neutralisation to remove joint angle variation during imaging. Repositioning was successful; no joint flexion variation was observed in the resulting model. The first principal component (PC) of morphological variation represented phalanx size in all fingers. Subsequent PCs showed variation in position along the palmar-dorsal axis, and bone breadth: length ratio. Finally, the models were interrogated to provide gross measures of bone lengths and joint spaces. These models have been published for open use to support wider community efforts in hand biomechanical analysis, providing bony anatomy descriptions whilst preserving the security of the underlying imaging data and privacy of the participants. The model describes a small, homogeneous population, and assumptions cannot be made about how it represents individuals outside the training dataset. However, it supplements anthropometric datasets with additional shape information, and may be useful for investigating factors such as joint morphology and design of hand-interfacing devices and products. The model has been shared as an open-source repository (https://github.com/abel-research/OpenHands), and we encourage the community to use and contribute to it.

## Introduction

Computational biomechanical modelling is a useful tool for surgical planning, clinical assessment, and testing of new devices for disease interventions and consumer products. Musculoskeletal (MSK) modelling and Finite Element Analysis (FEA) studies are commonly informed by parameters obtained from a variety of sources or are subject-specific. However, variability and uncertainty are present in factors such as patient geometry, material properties of tissues, kinematics and joint loading, and clinical outcomes. Therefore, to further broaden the interpretation of such studies’ outputs, it is possible to combine them with population-based approaches [1]. Biomechanical models can thus be varied systemically to describe individuals representative of a population, enabling model outputs to be interpreted relative to the trends observed in the wider population. This method also allows existing models to be applied to new individuals with relatively low expense, which may support clinical translation [2]. As encouraged by Saxby et al. [3], this approach relies on the research community to share models and technologies.

Statistical shape modelling (SSM) employs dimensionality reduction methods for characterising variations in factors including anatomic shape and tissue composition in a population [4]. The total variation in a training dataset is decomposed into a compact set of new variables. A statistical shape model provides a mean geometry of the training dataset and describes the shape variability as a series of ‘modes’. Some researchers size-normalise their data to separate the effects of size and shape variation [5, 6]. Population-based analysis of bone morphology using SSM may inform the design and testing of treatment devices and consumer products, as well as fundamental biomechanics studies. These models can be descriptive or predictive. Descriptive models [7–9] facilitate the study of shape characteristics and enable classification, measurement and investigation of any trends, clusters, or outliers within the dataset. Predictive models [10–13] can be used to study the relationships between shape and clinical or functional parameters and to reconstruct complete subject-specific geometries from incomplete data which is useful for informing other types of models.

This method has been employed to characterise the morphology of MSK structures including the mandible, residual limbs following amputation, and joints in the foot, knee and hip [6, 7, 14–16]. Considering the joints of the hand, statistical models have focused so far on the thumb, demonstrating morphological variation in the carpometacarpal (CMC) joint. Rusli and Kedgley [17] present the impact of morphology variation on joint instability across their study population, highlighting the significance of these findings in future CMC osteoarthritis studies. Schneider et al. [18] focused on characterising the morphological sex and age patterns amongst their study population of CMC joint bones. For example, they found that a female cohort had similarly shaped trapezium and first metacarpal bones as men. Although statistical models of the full hand's external anatomy have been reported [19]*,* there is no current SSM report on the interphalangeal joints found in the thumb or those found across the fingers, despite the prevalence of their degeneration [20] and scope to improve outcomes of their surgical interventions [21]. Few researchers have access to the anatomic data required for such analysis, and there is cost, inconvenience, and risk associated with CT or MRI scanning volunteers. Therefore, this study developed multi-body statistical shape models of the four fingers of the hand, providing models that can be published for open use whilst preserving the security of the underlying imaging data, to support wider community efforts in hand biomechanical analysis.

## Materials and Methods

### Construction of Statistical Model

Ethical approval was granted for Secondary Data Analysis of an existing dataset (ERGO Ref: 61718). The training dataset represented ten consenting participants (5F:5M, mean age 31 years, range 27–37 years), who were free from hand or wrist disease or injury and had been recruited for a finger motion capture and imaging study (IRAS Ref: 14/LO/1059) [22]. Each participant’s right hand was CT scanned (Discovery CT750 HD 128 scanner, GE Healthcare Inc. USA) with 0.3 mm voxels. Three scans were collected for each participant with the fingers in full extension, mid-flexion, and near-full-flexion. The resultant volume images were segmented to isolate the bony anatomy and meshed (ScanIP + FE, Synopsys Inc., United States). The resultant triangular surface meshes of the proximal phalanges (PP), medial phalanges (MP) and distal phalanges (DP) were imported into a MATLAB environment (The MathWorks, Massachusetts, USA). The data from the three positional scans were then aligned by the PP bones, and moved into a coordinate system [22] in which the origin lay at the PP centroid, and the sagittal plane was estimated from the movement of the MP bone during PIP flexion. The subsequent shape analysis used the meshes obtained from the fully extended scan (position 1) dataset, and scan positions 2 and 3 were used solely to estimate joint flexion axes for pose neutralisation, as described below.

A multi-body SSM pipeline, capable of computing the main modes of variation or ‘principal components’ (PCs) within the training dataset of three-dimensional surface meshes of the phalanges, consisted of three main stages.

To enable geometrical comparison between the training datasets, a single reference mesh was mapped to the surface meshes of each phalanx using an Iterative Closest Point (ICP) based non-rigid registration algorithm [23], establishing a nodal correspondence. The mesh with a length closest to the average of the dataset was selected as the reference mesh.

The registration error was calculated by computing the Root-Mean-Square-Error (RMSE) of the Euclidean distances between the target shape and the registered shape vertices. Additionally, a linear regression analysis was conducted to compare the mesh volumes before and after registration.

$$\text{RMSE }(x)=\sqrt{\frac{1}{N}{\sum }_{n=1}^{N}{\Vert {x}_{n}\Vert }^{2}}$$

where ${x}_{n}$ is the array of Euclidean distances computed using a k-nearest neighbour (kNN) search.

**Fig. 1:**
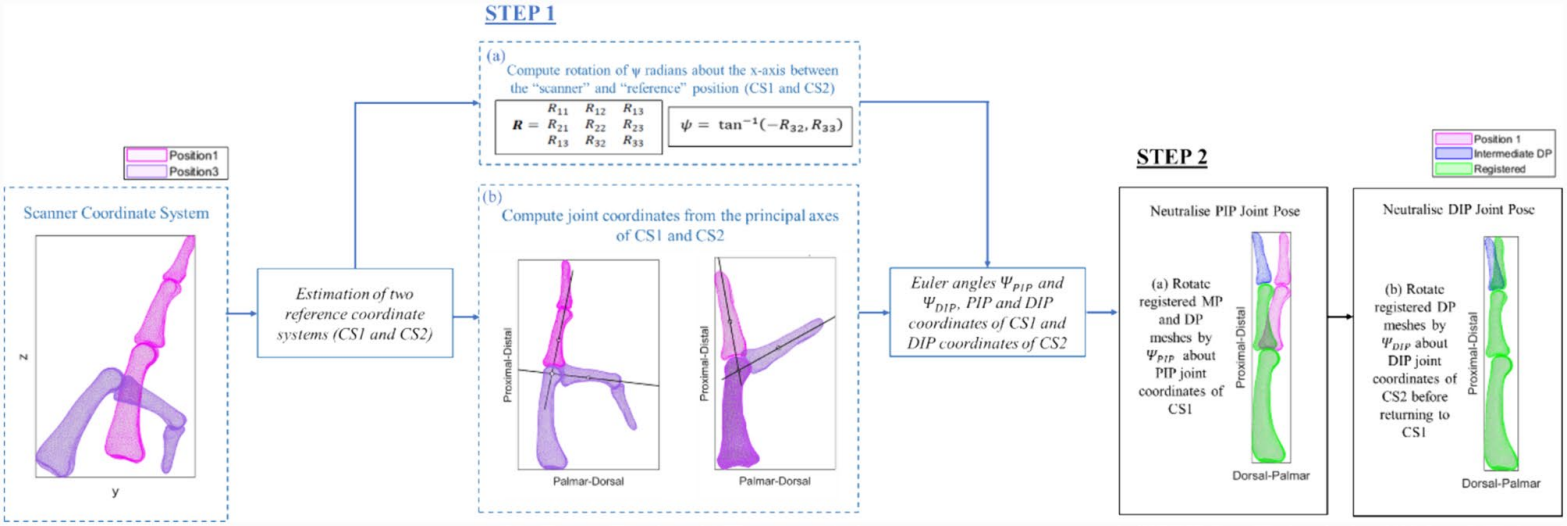
Flow diagram describing the joint pose neutralisation, to remove bone location variation generated during imaging. Step 1 computes the angles and centroids required for neutralisation. Step 2 involves the pose neutralisation of the PIP and DIP joint in the two reference coordinate systems (CS1 and CS2 respectively)

To assess how shape and scale variation could be captured in the statistical shape model with minimal influence of pose during scanning, the joint flexion in the “full extension” scan datasets was corrected (Fig. 1). Two reference coordinate systems were estimated to describe the bone positions relative to the PP and MP (CS1 and CS2, respectively) using the principal axes and centroids of the bone surface mesh vertices in their surface meshes, as reported previously [22].

The use of transformation matrices to compute the intrinsic sequence of rotations of the mesh vertices around the three different axes, known as a Euler rotation, is commonly used in biomechanics to study joint motion. For instance, Euler angles between the femoral and patellar coordinate systems were used to study the patellofemoral motion within a dynamic knee simulation model [24], and proximal interphalangeal (PIP) joint angles were extracted from an Euler rotation in a cadaveric study [25].

Singular value decomposition on vertex coordinates was used to calculate transformation matrices ${{\varvec{M}}}_{4\times 4}$, that describe the transformation of MP or DP from its position as scanned (‘scanner coordinate system’; i.e. Position 1 or 3) to the new reference coordinate system (CS1 and CS2). Euler angles ${\Psi }_{PIP}$ and ${\Psi }_{DIP}$ were used to estimate the PIP and distal interphalangeal (DIP) joint flexion-extension angles, and were calculated from the rotation matrix, ${{\varvec{R}}}_{3\times 3}$, extracted from ${\varvec{M}}$. These were used to align the joints into an approximately “neutral” pose, by rotation about estimated centroids of the PIP and DIP joints.

The PIP joint axis were estimated in CS1 by finding the intersection point between the MP bones’ long principal axes in the extended and flexed CT scans. This point was projected onto a plane through the PP and MP bone centroids in extension and full flexion, to which the joint axis was assumed to be perpendicular. The same process was then applied to the DP mesh’s principal axes to estimate the DIP joint axis in CS2.

Finally, each finger’s pose was neutralised by rotating the bone geometry by the angle measured between the proximal and distal bones’ principal axes in the “full-extension” scan.

PCA was applied to reduce the number of dimensions of the training dataset into a compact set of new parameters.

The three phalanx mesh files for each dataset were compiled into a column vector:

$${\varvec{x}} = {\begin{array}{ccc}[{x}_{1}& {y}_{1}& {z}_{1} \end{array}, \cdots , \begin{array}{ccc}{x}_{m}& {y}_{m}& {z}_{m}] \end{array}}^{T}$$

where $m$ represents the total number of nodes in the combined DP, MP and PP baseline meshes, and $x, y,$ and $z$ are the vertex coordinates.

A vector describing the mean finger shape was calculated by:

$$\overline{{\varvec{x}} }= \frac{1}{n}\sum_{i=1}^{n}{{\varvec{x}}}_{i}$$

where *i* represents each instance in the training dataset, and *n* the total number of instances.

PCA reduces the dimensionality of the training data by finding a new set of parameters, the PCs, which can be linearly combined to recreate the original 3*m* parameters that describe each instance *i* of the training data. The 3*m-by-3m* sample covariance matrix:

$${\varvec{S}}= \frac{1}{n-1}\sum_{i=1}^{n}\left({{\varvec{x}}}_{i}-\overline{{\varvec{x}} }\right){\left({{\varvec{x}}}_{i}-\overline{{\varvec{x}} }\right)}^{T}$$

is used to find these PCs. For a dataset where all the parameters describing variation between instances are uncorrelated, ${\varvec{S}}$ will be diagonal. It can be shown that the eigenvectors (${\boldsymbol{\varphi }}_{j}$) of ${\varvec{S}}$ can be used with a vector of weighting coefficients (${{\varvec{d}}}_{j}$) to reconstruct the training data [26]:

$${\varvec{x}}=\overline{{\varvec{x}} }+\sum_{j=1}^{c}{\boldsymbol{\varphi }}_{j}{{\varvec{d}}}_{j}$$

This constitutes a transformation of the original basis (3*m* parameters) into a new, much more compact, basis (the *c* PCs). These PCs are uncorrelated – their covariance matrix is diagonal – and ordered by decreasing variance (the first PC captures the dimension with the largest variability in the training data).

Singular value decomposition was performed over the analogous eigen analysis to undertake the PCA. The weighting coefficients (${{\varvec{d}}}_{j}$) used to generate new shapes, which show each PCs deviation from the mean, represent the 5th and 95th percentile range of the training dataset mode score and fit within the minimum and maximum mode scores bounds for each individual.

### Model Evaluation

To select a preferred model to publish open source, four statistical shape models were compared, to assess the relative influences of pose neutralisation and size normalisation, upon the resulting anatomic variance characterisation: (1) Original pose without scaling effects, (2) Original pose with scale effects, (3) Corrected pose without scaling effect, and (4) Corrected pose with scale effects.

Mode shapes were visualised by perturbing the mean shape from the 5th to 95th percentile range of the training dataset (i.e., by $\pm 1.654\sigma$), one PC at a time. To observe the potential similarities in variation between fingers, linear regression analysis was performed to assess the mode score correlation. Mode scores for each finger’s PCs were compared to the corresponding PC of the index finger. To describe the training datasets and resulting model shapes, the length of individual bones and the whole finger were calculated, along with an estimate for the joint spacing. A Shapiro-Wilk test indicated that these measures were normally distributed for the training shapes (p > 0.05) so parametric statistics could be used. The joint space was calculated using a kNN search with k = 1, to find the Euclidean distance from each vertex on the joint’s proximal surface to the distal surface, from which the median was calculated.

Four model performance measures are commonly used. These include model compactness, accuracy, generalization and specificity [27, 28]. This report presents compactness, accuracy and generalization test. However, we elected not to perform a specificity test because the generation of virtual individuals was outside the scope, owing to the relatively small training dataset.

Compactness: To observe how much variation is captured within the resultant PCs, the compactness was calculated and is defined as the cumulative variance of the *m*th mode or PC, used in the shape reconstruction [28]:

$$C(PC)={\sum }_{m=0}^{PC}{\uplambda }_{m}$$

Accuracy: The average RMSE was calculated to observe the variation in mean shape reconstruction with a limited training dataset [27]:

$$\text{RMSE}=\sqrt{\frac{1}{n}{\sum }_{i=1}^{n}{\Vert \overline{{\varvec{x}} }-{{\varvec{x}}}_{i}{\prime}(PC)\Vert }^{2}}$$

where $\overline{{\varvec{x}} }$ is the mean shape constructed with the complete dataset and the reconstructed mean ${{\varvec{x}}}_{i}{^{\prime}}$ is a linear combination of the training datasets.

Generalization: To evaluate the quality of the constructed statistical shape model, a Leave-One-Out cross-validation test was performed, calculating the average deviation between the resultant statistical model’s mean and mode extremes $(\pm 1.654\sigma )$ shapes and those reconstructed by removing one dataset from the PCA calculation. [27, 29].

$$\text{Average\,\, Deviation }=\frac{\sum \sqrt{\frac{1}{n}{\sum }_{i=1}^{n}{\Vert {\varvec{X}}-{{\varvec{X}}}_{i}{\prime}(PC)\Vert }^{2}}}{n} = \frac{\sum RSME}{n}$$

where ${\varvec{X}}$ is the shape constructed with the complete dataset from the statistical model and the shape ${{\varvec{X}}}_{i}$ constructed using (n-1) training datasets.

## Results

### Model Evaluation and Selection

The registration error across all fingers was less than 0.4 mm for each phalanx mesh, and the registered bone volumes were within 3% of the target bone shapes (R^2^ = 0.95).

**Fig. 2:**
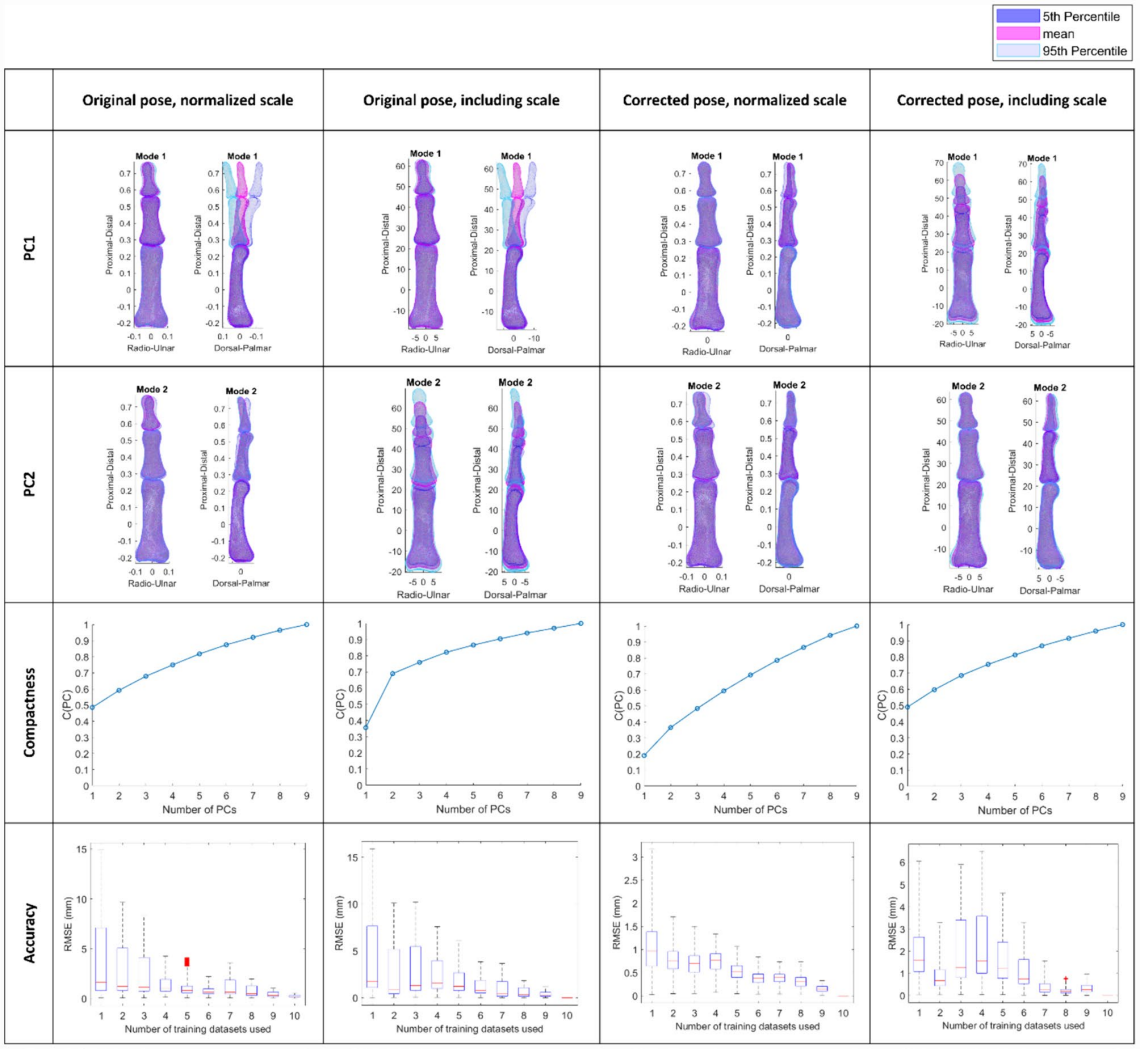
Impact on pose neutralisation and normalising scale on resultant principal components. Note for normalized shapes (column 1 and 3): since the proximal phalanx’s centroid lies at [0 0 0], the mean (against which dimensions are normalized) has a full scale length of one but is represented between approximately − 0.25 and 0.75 along the Proximal-Distal axis.

Nine principal components (PC1 – PC9) representing the main morphological variation were found for the three phalanges of the index, middle, ring and little fingers. Observation of compactness for models with and without size normalisation and pose neutralisation indicated that over 75% of shape variability was captured within the first four modes and 90% within seven modes for three of the model types (Fig. 2). The model constructed with corrected pose and normalized scale was less compact with only 59% of shape variability captured in the first four modes. The model constructed using pose neutralisation and full scaling had the combination of high compactness and small error in mean shape reconstruction, so all subsequent results (Fig.s 3-7 and Tables 1, 2 and 3), are based on using the "Corrected Pose, including scale" model.

**Fig. 3:**
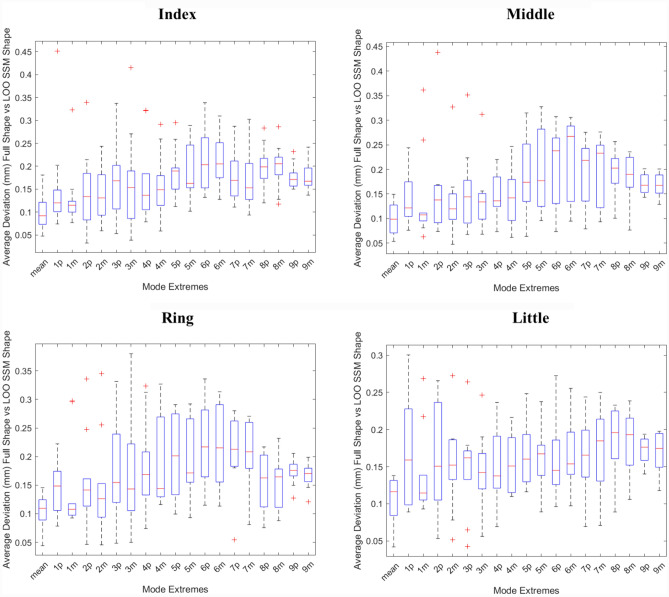
Deviation between the mean and mode extreme shapes generated using n = 10 (Full Shape) and the mean and mode extreme shapes (p represents minimum shape and m represents maximum shape for PC1 – PC9) generated when one dataset is removed (n = 9, LOO SSM Shape)

**Table 1:** R^2^ and p-value from linear regression analysis of the PC weights of index finger compared to the PC weights of the middle, ring and little fingers.

	Finger	R^2^
PC1	Index vs Middle	0.938*
	Index vs Ring	0.892*
	Index vs Little	0.869*
PC2	Index vs Middle	0.771*
	Index vs Ring	0.760*
	Index vs Little	0.062
PC3	Index vs Middle	0.373
	Index vs Ring	0.106
	Index vs Little	0.200
PC4	Index vs Middle	0.429*
	Index vs Ring	0.013
	Index vs Little	0.064

* denotes p < 0.05

**Table 2:** Average index finger bone lengths (in mm) from training dataset and average, 5th and 95th percentile index finger bone lengths (in mm) from first principal component statistical shape model-generated shapes

	Mean (5th − 95th percentile range) Bone Lengths, mm			
	DP	MP	PP	Total
Training datasets	17.5 (15.3-19.7)	24.6 (22.7-26.8)	40.9 (37.7-43.9)	83.0 (75.7-90.4)
Statistical Model PC1	17.4 (15.6-19.5)	24.8 (22.0-27.7)	41.2 (37.5-45.0)	83.5 (75.1-92.2)

**Table 3:** Average finger DIP and PIP joint space (in mm) from first principal component SSM-generated shapes

	Mean (5th-95th percentile range) Joint Space, mm	
	DIP	PIP
Training datasets	2.2 (1.9–2.5)	2.5 (2.3–2.9)
Statistical Model PC1	2.2 (2.1–2.3)	2.5 (2.5–2.6)

The Leave-One-Out test highlighted a minimal deviation (<0.2 mm in the mean and < 0.5 mm in the mode shape extremes) between the resultant statistical shape model and the reconstructed model (Fig. 3).

### Description of the Selected Model

**Fig. 4:**
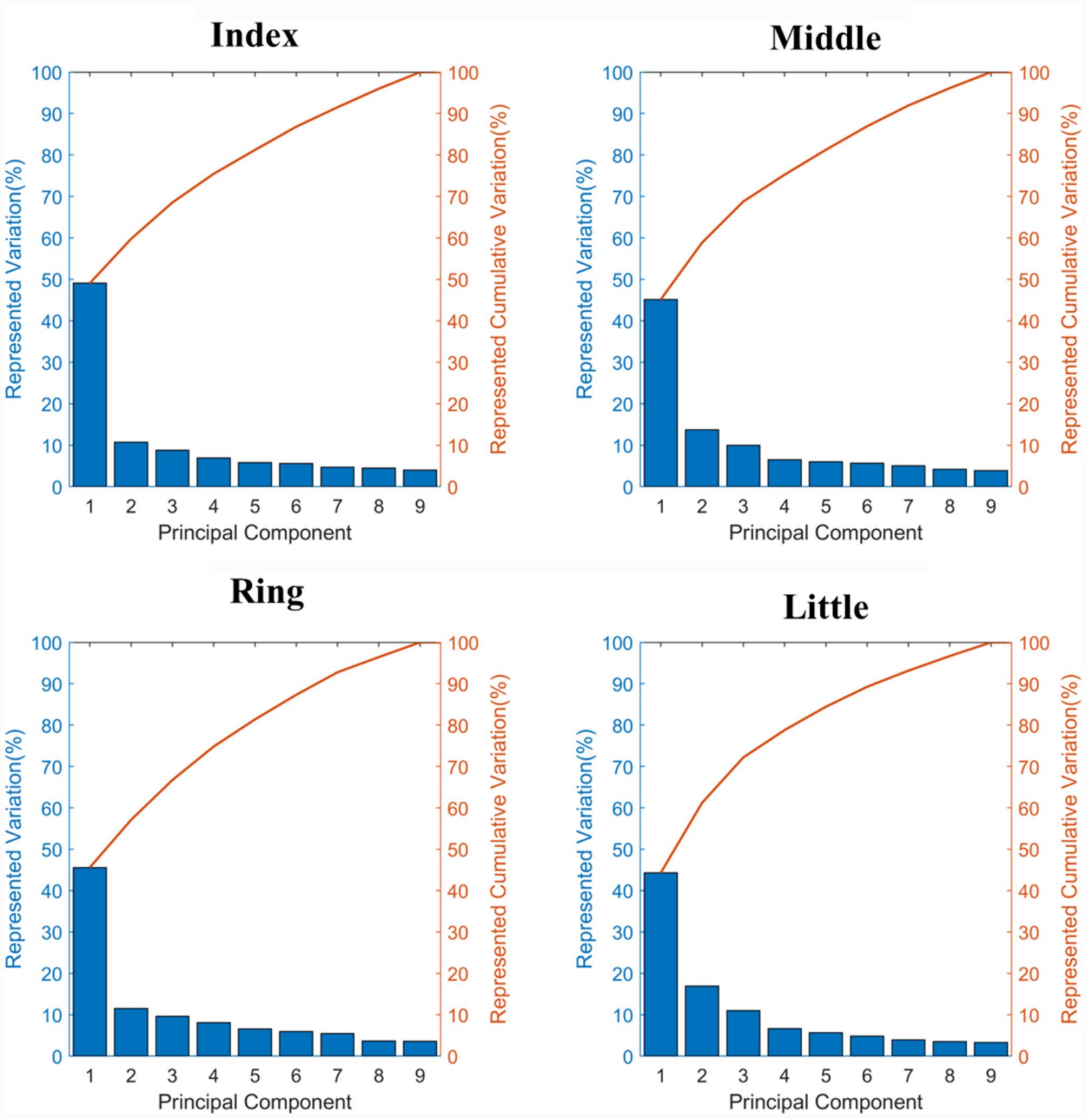
Variance (bar) and cumulative variance (line) captured by all PCs for index (A), middle (B), ring (C) and little (D) finger

The first four PCs across all fingers accounted for over 75% of the total variation within the training population, of which over 45% was attributed to the first PC representing phalanx size for all fingers (Fig. 4).

A strong correlation was observed (Table 1) between PC1 of the index finger and PC1 of the remaining fingers (R^2^ > 0.869). The PC1 mode score correlation agrees with the visualization of PC1 for all fingers, whereby size was the dominating PC. The R^2^ values for subsequent mode scores were lower (0.01 < R^2^ < 0.77), indicating that the variation described by each mode was not always the same.

**Fig. 5:**
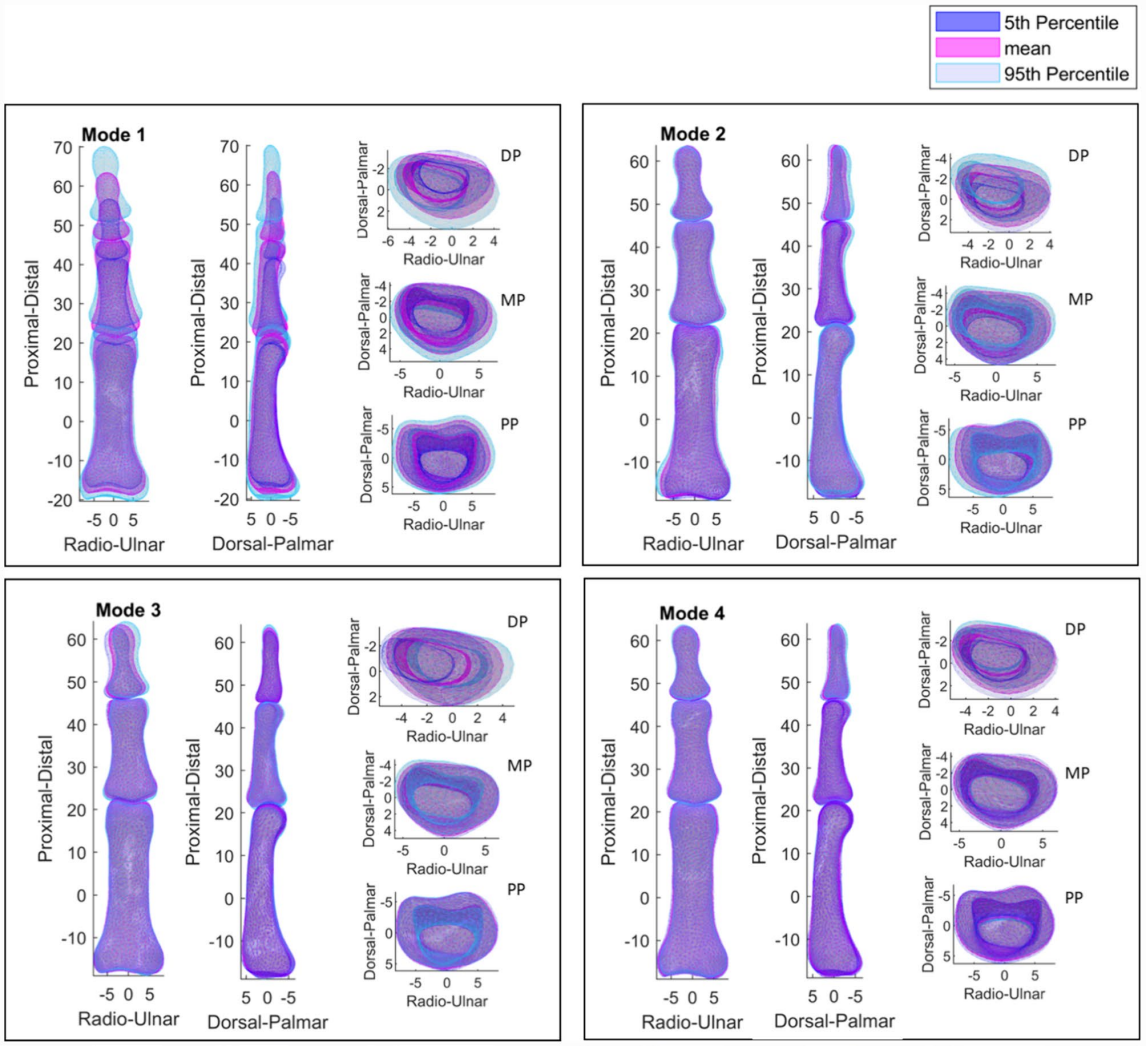
Index Finger PCs: First principal component showing variation in bone size, the second principal component showing positional variation along the Palmar-Dorsal axis, the third principal component showing orientation variation and the fourth principal component showing variation in bone breadth

Visual inspection of the PCs (Fig. 5) suggests that PC1 presented variation in bone size, PC2 described positional variation of the bones along the palmar-dorsal axis, PC3 represented variation in ab/ad-duction in the dorsal-palmar plane and PC4 indicated variation in bone breadth.

Gross measures were extracted from the mean and 5th-95th percentile range in shape of PC1 and compared to the same measures taken directly from the training datasets (‘CT’) to illustrate the model’s ability to represent the population’s size range. (Fig.s 6 and 7). This indicates that the PCA method was able to extract the size variation within the training dataset, predominantly within a single mode (PC1).

**Fig. 6:**
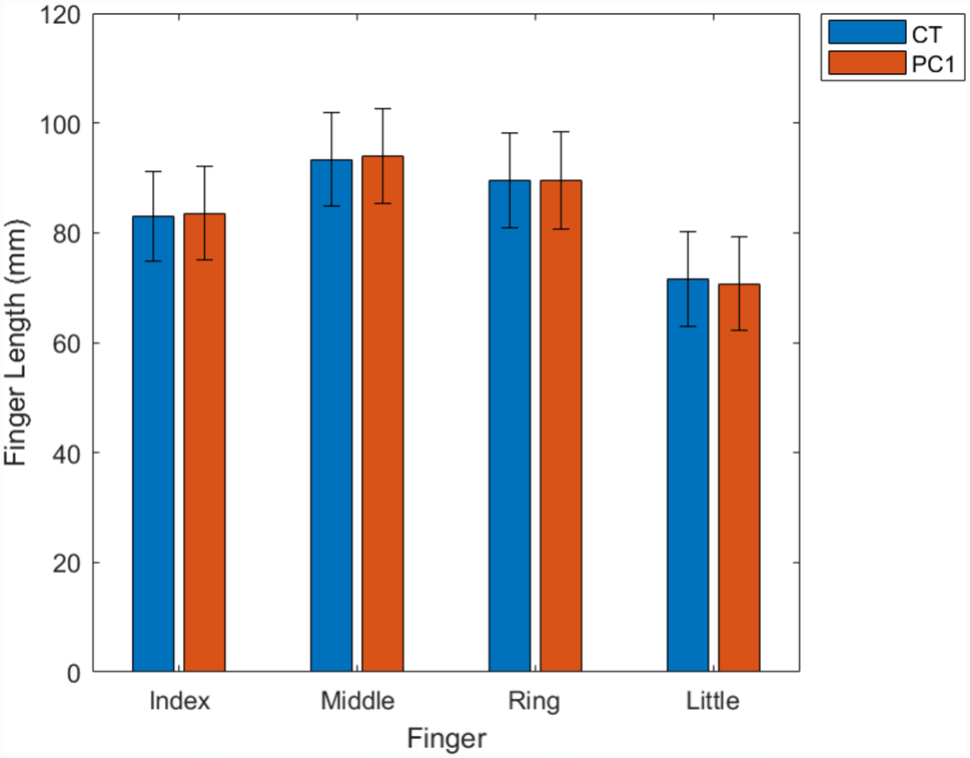
Average and 5th to 95th percentile range (error bars) finger length in mm across training datasets and PC1 shape instances

**Fig. 7:**
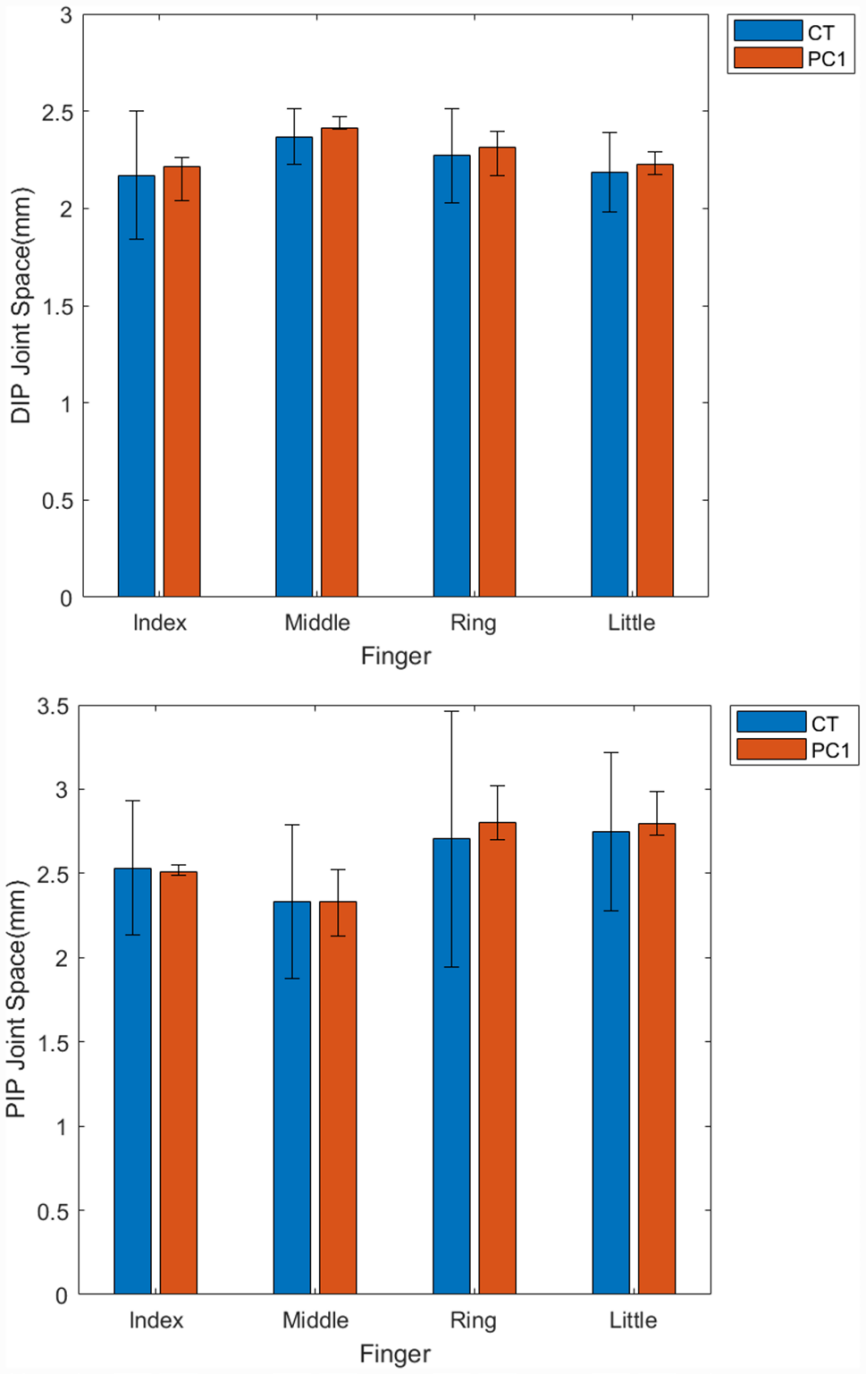
Average and 5th to 95th percentile range (error bars) for DIP (top) and PIP (bottom) joint space in mm across training datasets and PC1 shape instances

Conversely, though the mean joint space from the statistical shape model was in close agreement with that of the training dataset, the variance in this measure was greater than that contained within PC1 indicating that it is distributed across subsequent modes. The training datasets indicated that finger length and joint space were not associated (R^2^ = 0.1 and 0.007 for DIP and PIP, respectively).

## Discussion

This study presents a pipeline for generating a statistical shape model of the fingers. It has novelty in being trained using medical imaging from living participants, free from hand or wrist injury, and the process estimates the removal of pose variation during imaging, to maximise the observable size and shape variation from a small dataset.

Scale is a common mode of variation and dominates as the largest portion of morphological variation amongst a population. It is argued to largely contribute to morphological variation, i.e. an increase in the size of a structure suggests an increase in the skeletal dimensions such as bone shaft, breadth and articulating surface. Whether scale is an important PC is at the discretion of the user, however for the extraction of gross measures from each PC such as bone length and joint space, the pose corrected model with scale effects was used for further analysis and publication on the open-repository. Bone lengths were extracted to illustrate this variation within our training dataset, and these were significantly larger than finger lengths reported in previous studies [30, 31] (i.e. mean index finger length for right hand [30] were 79.7 mm ± 5.1 mm for male participants and 73.6 mm ± 5.0 mm for female participants whereas for the training datasets, the mean index finger length for right hand was 86.6 mm ± 3.4 mm for male participants and 79.3 mm ± 2.7 mm for female participants). However, these may not be completely comparable measures, as the present data are direct bone length measures whereas the cited prior data are estimated finger segment lengths based on fingertip to finger-palm crease distance and are inclusive of distal soft tissue. All considered, our method remains objective and repeatable across different fingers and datasets and may avoid subjectivity of external measurements.

Whilst sizes are likely to be important for anthropometrics and ergonomics, in other research questions, shape may be of more interest. For example, Bruse *et al.* [5] excluded size effects when applying a statistical shape modelling framework to extract 3D shape biomarkers of repaired aortic coarctation arches. Similarly, Cerveri *et al.* [16] were solely interested in knee joint instability and therefore selected non-size-related PCs, such as the height of the femoral and tibial shafts, and the curvature of the femoral shaft and in the frontal plane. The present study provides researchers with the opportunity to study both size- and shape-dominated phenomena.

According to Wang and Shi [28], a compact statistical model represents the population variance with a small number of PCs. The number of modes retained in a model is often simply determined by selecting those which cover a percentage threshold of the total variance (most popularly a 95% variance threshold). Limited by the training dataset size, we would need to retain all the modes to achieve this. This limitation is discussed by Mei *et al.* [32] who show that this retention method is highly dependent on sample size, recommending that mode retention should ideally correspond to genuine anatomical variation.

The authors believe that these are the first publicly available statistical shape models of the fingers’ skeletal anatomy generated from living participants. The nearest comparable dataset is that generated by Van Houtte *et al.* [19] who present an articulation-based registration method for three-dimensional meshes of human hands. SSM was successfully applied to 100 human hand shapes and provided an insight into the anatomic variation of the lower arm and hand. In their study, they mainly present the effectiveness of the proposed registration framework for the design of well-fitting products rather than discuss the skeletal variation.

In a wider context, with the varying sizes, positions and orientations extracted from the PCA, these statistical shape models have application in several areas including the design of products that account for diversity in anthropometrics like orthopaedic implants and consumer devices, and studies looking at how nature of bone/geometry can affect propensity for conditions such as arthritis. Patient perspective is increasingly becoming a valued part of this process, and to capture this, three members of the public living with a hand joint condition were consulted to discuss their perspectives on the usability of a computational model of the fingers. Whilst the data used to construct the model was collected before their involvement, contributors expressed that generating a model that could be accessed by the wider community could also be a useful educative tool for members of the public and clinicians as well as for researchers. A large emphasis was put on the idea that “one size does not fit all” and that a potential model should address the variability between individuals. In addition, they advocated for such a model to be made available for not only the public but for practitioners and the wider research community, especially for those designing products for the hand. As potential end-users, the public contributors believe that it is important that researchers consider how these products or interventions may perform on different hand and finger shapes and sizes. Public contributors also suggested that future iterations of a statistical model could include a focus on including datasets from other pathologies such as osteoarthritic datasets.

This study is limited primarily by a small training dataset representing a homogeneous population of young, healthy participants, mainly working in tertiary or quaternary sectors. This might represent a portion of the UK population but may not describe those who use their hands more heavily, such as those who work in primary and secondary sectors. In addition, the exemplar model produced in this study does not capture the influences of pathology or surgery upon the condition of the soft and hard tissue. These models solely focus on the morphology of the phalanx skeletal structure. If one were to use these models to study the kinematics and kinetics of the hand, more data would be needed, hence why they have been made available for contribution and collaboration. The inclusion of additional datasets is non-trivial, however, because the phalanges should be aligned neutrally, which cannot be guaranteed at the point of imaging. This workflow corrected the phalanx alignment using estimation of the position and angle of the DIP and PIP joints using scans of the participants’ hands in at least two positions, which is not a standard clinical imaging protocol. However, it may be possible to expand the model’s training dataset from single-position CT images by further development of functional joint axis estimation using motion capture [33]. Further, pose neutralisation only corrected PIP and DIP flexion-extension, assuming that abduction-adduction and internal-external rotations in the full-extension scans were relatively small, and this may be valid because the flexion corrections averaged 1.0° and ranged from ± 16° for all except one dataset (23°).

This paper presents a process for generating population models of the finger and shares an exemplar dataset open source (https://github.com/abel-research/OpenHands) for community use. The shared model describes a small, homogeneous population, and assumptions cannot be made about how it represents individuals outside the training dataset. However, such a model can supplement gross anthropometric datasets with additional shape information, and if trained with additional CT images the model may be of use for investigating factors such as joint morphology, and for the design of hand-interfacing devices and products. We encourage the community to use it and to contribute.
